# Can fostering posttraumatic growth prevent burnout and promote resilience in future nurses?

**DOI:** 10.3389/fpubh.2025.1665351

**Published:** 2025-09-30

**Authors:** Jae-Chang Sim, Dayoung Lee, Jubeen Park, Sun-Young Im

**Affiliations:** Department of Psychology, Hallym University, Chuncheon-si, Republic of Korea

**Keywords:** burnout, posttraumatic growth, resilience, nurses, psychological intervention

## Abstract

**Objective:**

This study aimed to develop and validate a psychological support program incorporating psychological factors that promote PTG, in order to prevent burnout and enhancing resilience in future healthcare workers in preparation for infectious disease outbreaks such as the COVID-19 pandemic. The program was developed with consideration to the characteristics of nurses. Since nurses, particularly, suffered from severe burnout during the COVID-19 pandemic, the efficacy in this study was tested in nursing trainees.

**Method:**

Based on a review of related literature, we developed a program consisting of eight sessions. The content of the program includes physical and emotional recognition, finding meaning in stressful events, understanding and reflection on posttraumatic growth (PTG), promoting happiness and finding value, and inspiring hope. To test the effects of the program, participants were divided into control and intervention groups. PTG, resilience, burnout, emotion processing, deliberate rumination, and mental wellbeing were measured pre-intervention, post-intervention, and at later follow-up.

**Results:**

Using a repeated-measures analysis of variance, the treatment group showed increased PTG, resilience, mental wellbeing, and emotion processing after the intervention compared with the control group, and this increase was maintained in the follow-up. Although the effect on burnout was not statistically significant, there was a trend for relatively decreased burnout in the treatment group.

**Conclusion:**

These results demonstrate the potential effectiveness of psychological program in promoting healing and growth to support healthcare workers’ mental health. By fostering PTG and resilience, the program offers practical benefits for preventing future stress and burnout in healthcare settings.

## 1 Introduction

The Coronavirus disease 2019 (COVID-19) pandemic highlighted the importance of not only infection control policies, but also psychological protection from the effects of pandemics. Research on the psychological effects of infectious disease outbreaks has accumulated since the 2002 SARS outbreak and the 2013 MERS outbreak. The recent COVID-19 pandemic has had long-lasting effects in terms of the affected regions, scale, and duration, further emphasizing the need to prepare against future infectious disease outbreaks. Preventive measures can help minimize the possible psychological after-effects in the event of similar disasters in the future. Notably, healthcare workers, who are the first responders on the front lines where they are most exposed to the risk of infection, deserve special attention. Healthcare workers experienced a sharp increase in work burden due to the COVID-19 pandemic (1–6). Burnout and mental health problems in healthcare workers require attention and active management not only for their personal wellbeing, but also because disease and work absence in healthcare workers can directly affect infection control systems and healthcare services (7, 8).

Burnout is a typical symptom that might be experienced by healthcare workers during a novel infectious disease outbreak, and is defined as exhaustion and inability to perform work, or disengagement and unwillingness to perform work (9). They may also show negative, cynical, or indifferent attitudes towards others (10). As the quantity and quality of work demanded of an individual increases, if they do not have the opportunity to recover and are overly stressed, they may experience burnout (11). Hence, during novel infectious disease outbreaks, wherein healthcare workers’ workload increases geometrically, their risk of burnout also increases.

Indeed, healthcare workers worldwide have been found to experience considerable stress and mental health issues, including posttraumatic stress disorder (PTSD), anxiety, depression, insomnia, and burnout, during novel infectious disease outbreaks. These symptoms have been observed in both the acute phase of an outbreak and after the end of the outbreak (8). During the COVID-19 pandemic, among healthcare workers, nurses especially had a high prevalence of PTSD (6) and high levels of burnout due to heavy workloads and psychological difficulties (12). Since the loss of nursing staff due to burnout significantly impacts patients who require healthcare support, it is essential to prepare measures to prevent burnout in nurses.

Therefore, we explored the factors that could prevent burnout in nurses. In particular, we hypothesized that posttraumatic growth (PTG) could act as an effective buffer against burnout. PTG refers to the positive psychological change experienced by people as a result of fighting through challenging situations in their lives (13). According to a study on PTG in nurses during the COVID-19 pandemic (14), nurses experienced moderate or severe psychological stress while working, but stress due to COVID-19 could be redirected to positive change through a process of deliberate rumination. Research has reported a negative correlation between PTG and burnout in frontline healthcare workers (15), and higher levels of PTG have been shown to correlate with lower levels of depression and anxiety (16) and reduced experience of burnout at work (17).

In a preliminary study (blind), we explored variables with significant effects on burnout and PTG in 247 nurses in South Korea. We identified emotional expressivity, deliberate rumination, and cognitive emotion regulation as significant predictors for PTG and burnout. PTG acts as a mediator between these variables and decreased burnout. These results suggest that deliberate rumination, emotional expression, and adaptive cognitive emotion regulation could be used to alleviate or prevent burnout in nurses, and could promote PTG from the negative effects of COVID-19. Deliberate rumination occurs when the individual engages in the cognitive task of trying to understand the causes for the events they experienced in an attempt to adapt to changes in their life caused by trauma (81). Several empirical studies have provided support for the idea that deliberate rumination is an important predisposing factor for PTG (18, 19). Trauma survivors can experience severe emotional distress, and their outcomes for recovery and growth can differ depending on how they cope with that emotional distress (20). Emotional processing, which is the process of recognizing and accepting distressing emotions, helps to promote deliberate rumination to find meaning in traumatic experiences (21, 22, 82).

Meanwhile, several studies have focused on resilience as a protective factor for mental health in nurses. Resilience is defined as a personal characteristic that regulates the negative effects of stress and helps with post-stress adaptation, and as the ability to successfully cope with change or danger (23). Previous studies have reported that resilience helped nurses perform their work better during the COVID-19 pandemic and reduced the prevalence of burnout, while also improving mental wellbeing (24–30). Resilience can also help reduce nurses’ post-traumatic stress and burnout by adapting to their environment, thereby enabling positive experiences such as PTG (27, 31, 32). Based on the aforementioned evidence, developing interventions that can improve resilience is essential to alleviate negative effects on nurses’ mental health (24).

The psychological support programs that have been developed for healthcare workers to date are limited because the target symptoms do not comprehensively include emotional, cognitive, and physical factors. For example, Jang and Kim (33) focused on cognitive tasks, such as self-pity, understanding of mindfulness, and learning the difference between self-love and pity, while the forest healing program run by the National Trauma Center (34) emphasized physical and emotional rest. However, people suffering from burnout complain of difficulties in emotional, cognitive, and physical aspects (35), highlighting the need for a multidimensional approach. Given that one of the characteristics of people suffering from burnout is loss of motivation, these programs should also include motivation. Accurately recognizing one’s own condition is the first step of treatment. In other words, understanding the nature of burnout can improve treatment motivation and effectiveness; hence, basic psychological education is essential (33, 36, 37). However, the development and implementation of actual psychotherapy programs are still lagging.

Therefore, the program in this study was designed to include psychological education to help people suffering from burnout understand their own conditions without imposing a secondary burden. Moreover, existing programs for healthcare workers focus on “recovery” by preventing and alleviating occupational burnout, than promoting personal growth. In a study by Yoon (38), the H.A.T. program had no significant effect on personal growth or purpose in life as subfactors of psychological wellbeing in clinical nurses. Therefore, this study aimed to go beyond the limited goals of minimizing distress and maladaptation by emphasizing growth and increasing positive personal resources.

In other words, our program aimed not only to help healthcare workers recover from the distress caused by infectious disease outbreaks but also to provide an opportunity for PTG by directing this distress toward qualitative, positive changes. Specifically, we aimed to develop and validate a program suited to healthcare workers in South Korea to prevent burnout and promote PTG. As a pilot study to test the program’s effects, the program was implemented in trainee nurses enrolled in a 4-year university nursing degree. The participants’ mental health-related variables were measured and statistically compared at three time points: before the program (pretest), immediately after the last session of the program (posttest), and 1 month after the program (follow-up). To test whether changes over time reflected the effects of the program, the participants’ mental health-related variables were compared with those of a wait-list control group. Based on a review of prior studies, burnout, mental wellbeing, resilience, and PTG were employed as the dependent variables. To investigate the process of promoting PTG, we measured the cognitive factor of deliberate rumination and the emotional factor of emotional expression, which are important intermediate variables for achieving PTG in people who have experienced severe stress or trauma.

The main hypotheses in this study were as follows. The first hypothesis was that the treatment group would show decreased burnout and improved emotional wellbeing, PTG, resilience, deliberate rumination, and emotional processing compared with the control group after completing the program. The second hypothesis was that the changes in measured variables in the treatment group compared with the control group would be maintained even at follow-up.

## 2 Materials and methods

### 2.1 Program development

To develop the program for this study, we referred to the four-stage program development model suggested by Kim et al. (39). First, the program was planned, and the program goals and theoretical model were set. Second, the content of the program was reviewed theoretically, and the program content was composed to suit the study goals. To select activity elements for the program, we reviewed previously developed programs and literature related to burnout and PTG (e.g. (18, 36, 40–44)). Either activity elements extracted from the literature review were suitably amended, or new activity elements were developed. Third, pilot tests of the initial program were conducted, and the program was modified after assessment and review. The first pilot test was performed by the researchers, and then a second pilot test was performed including nursing students with experience of hospital practice. Based on the results, some of the content was adjusted. Fourth, the modified program was administered to the target participants and assessed using various methods. This was the main implementation stage to test the effects of the program.

Several important considerations should be taken into account when developing psychological intervention programs. First, considering the interpersonal dynamics involved in programs that include group counseling (45), relatively easy and comfortable activities were placed at the beginning of the program to focus on building trust among the group, while more complex activities were placed toward the later sessions. Second, the program’s activity elements were constructed according to the three-stage model of trauma treatment (46). (1) The safety and stabilization stage mostly includes activities for forming rapport between participants and regulating burnout or stress symptoms (e.g., relaxation training). (2) In the trauma memory processing stage, techniques are implemented to process traumatic memories and prevent burnout symptoms. (3) In the reconnection stage, the participants find meaningful relationships and everyday activities in reality so that they can integrate these into their own lives and grow. Finally, the participants think deeply about advice and recommendations from the perspective of PTG. Wortman (47) warned that therapists must not show expectations that PTG is universal and that all patients must experience PTG, since this can burden them. Indeed, PTG is neither inevitable nor universal; hence, caution is necessary not to imply that people who do not experience growth are somehow deficient (48). Throughout the program, the researchers continually exercised care not to burden participants with the feeling that they must experience PTG.

### 2.2 Program schedule and composition

The program was structured into eight sessions. The content was divided into five topics, and there were 19 activities, each corresponding to a specific topic: (1) program orientation and formation of group relationships, (2) physical and emotional awareness and expression, (3) finding meaning in stressful events, (4) finding happiness and value, and (5) inspiring hope and debriefing. Although the program was structured into eight sessions, to account for the schedules of the participants, who were students, two sessions each were combined into a 160-min slot, once per week. The detailed contents of the program are shown in Supplementary material. The program was implemented by two of the researchers who had professional qualifications in counseling and clinical psychology, and the other researchers helped with running the program.

The goal of first topic is for the group members to understand the contents and method of the group program and form rapport with each other and with the group leaders. In the second topic, participants enhance their physical and emotional awareness through various activities, including mindfulness. In the third topic, participants receive psychoeducation on stress and burnout, and engage in activities to deliberate on their experiences of burnout and find meaning in them. The activities in the fourth topic are designed to encourage participants to explore their happiness and values and to set directions for their lives. The participants plans for their lives based on what they have learned and experienced in the program so far. A more detailed description of the program is provided in the Appendix.

### 2.3 Participants and measurement process

The participants were 36 nursing students in clinical training enrolled at a 4-year university in Gangwon-do who voluntarily consented to participate in the study. All participants were provided compensation, with the amount varying depending on the number of sessions and questionnaires completed. The details of participant recruitment and selection, the study procedure, and compensation were explained in advance, and all the study procedures were approved by the institutional review board at the authors’ affiliated institution before commencing the study (HIRB-2023-065).

**Figure 1:**
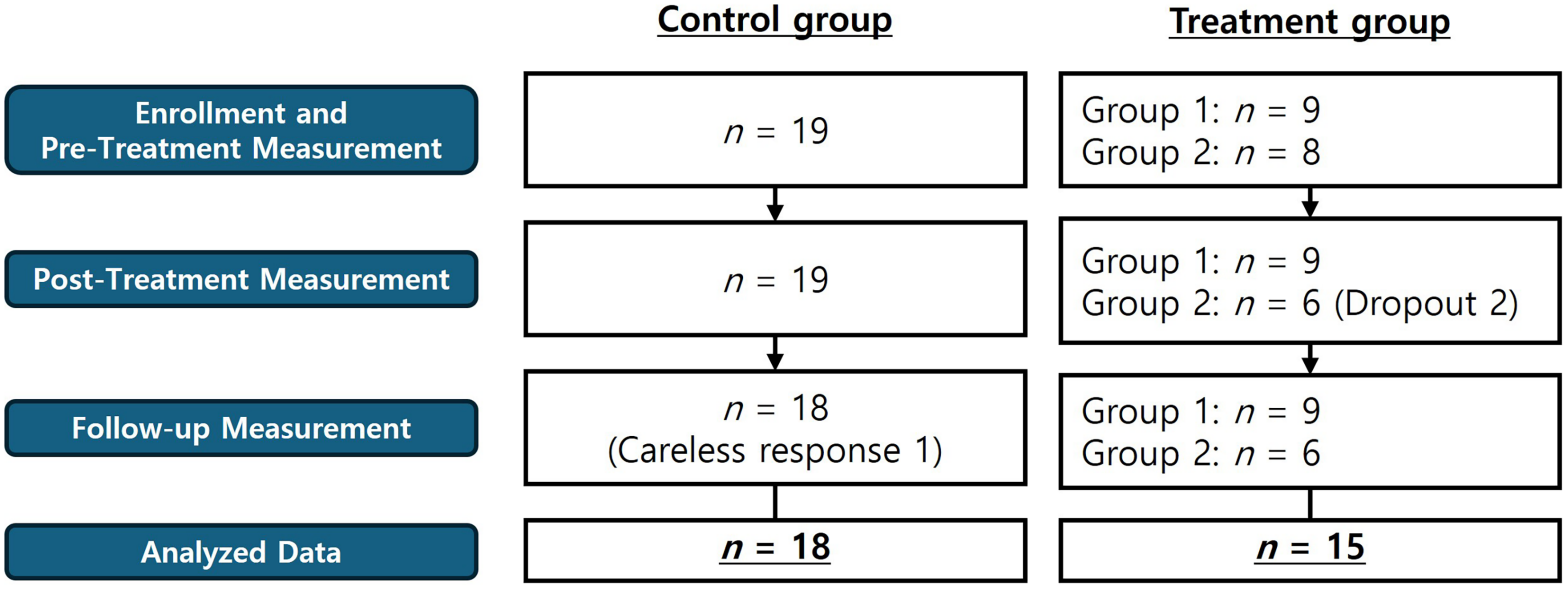
Research procedure and participants.

Figure 1 shows the participants per group and the study procedure. The participants were allocated based on their own voluntary intentions to either the treatment (n = 17 persons) or control groups (n = 19 persons). The treatment group was divided into two groups for the treatment sessions. The control group participants, after completing the follow-up questionnaire to test the effects of the program, were offered the chance to receive the main program or psychological education, as they wished. In treatment group 1, all nine participants successfully completed all sessions with no dropouts, while there were 2 dropouts in treatment group 2. Of the 19 participants in the control group, one person was excluded from the analysis due to insincere responses. A total of 33 persons were included in the final analysis. The mean age of the participants was 21.4 years old (SD = 2.06). Of the 33 participants, 28 were female, and 13 were religious. More detailed demographic information is provided in the Appendix.

Measurements for both groups were taken at three time points: pretest, posttest (4 weeks after the pretest measurements for the control group), and at follow-up 4 weeks after the end of treatment (4 weeks after the posttest measurements in the control group). At the pretest measurements for all variables, the participants were instructed to recall their experiences of hospital practice during the COVID-19 pandemic when responding.

### 2.4 Instruments

#### 2.4.1 Posttraumatic growth inventory-extended (PTGI-X)

This scale was originally developed by Tedeschi et al. (49) to measure individuals’ perceptions about positive changes after a traumatic experience. In this study, we used a version adapted to suit Korean culture and validated by Im (50). The scale comprises 25 items and is divided into five subfactors: new possibilities, relating to others, personal strength, spiritual-existential change, and appreciation of life. All items were scored on a 6-point Likert scale The internal consistency (Cronbach’s α) in this study was 0.94 for all items and 0.75–0.87 per subfactor.

#### 2.4.2 Burnout assessment tool (BAT)

This is a self-report scale that was originally developed by Schaufeli et al. (51) to measure occupational burnout, and was adapted and validated in Korean culture by Cho (52). The Korean version comprises 22 items, which is one less than the original version, and all items are scored on a 5-point Likert scale. The original version measures four core symptoms (exhaustion, psychological distance, cognitive impairment, emotional impairment) and three secondary symptoms (depressed mood, psychological distress, psychosomatic complaints), but the Korean version has only been validated for the core symptoms (52). The internal consistency (Cronbach’s α) in this study was 0.94 for all items, and 0.80–0.92 per subfactor.

#### 2.4.3 Mental health continuum-short form (MHC-SF)

This is a self-report scale originally developed by Keyes et al. (53) to measure mental wellbeing, and adapted to Korean culture, shortened, and validated by Lim et al. (54). It comprises 14 items, each scored on a 6-point Likert scale, with higher scores indicating higher levels of mental wellbeing. It has three subfactors: emotional, social, and psychological wellbeing. The internal consistency (Cronbach’s α) in this study was 0.92 for all items, 0.93 for emotional wellbeing, 0.66 for social wellbeing, and 0.86 for psychological wellbeing.

#### 2.4.4 Event-related rumination inventory (ERRI)

The ERRI was originally developed by Cann et al. (55) to measure intentional rumination, and the Korean version was validated by Ahn et al. (83). This is a self-report scale, with items scored on a 4-point Likert scale. Of the intrusive and deliberate rumination subfactors, in this study, only the 10 items for deliberate rumination were used. The internal consistency (Cronbach’s α) for the 10 deliberate rumination items in this study was 0.95.

#### 2.4.5 Emotional approach coping scale-16 items (EAC-16)

This scale was originally developed by Stanton et al. (56) to measure the level of emotional processing. It was adapted and validated in Korean by Kang and Yang (57). Of the subfactors, only the eight items for emotional processing were used in this study. All items are scored on a 4-point Likert scale, with higher scores indicating higher levels of emotional processing. The internal consistency (Cronbach’s α) for the eight emotional processing items in this study was 0.92.

#### 2.4.6 Connor-Davidson resilience scale (CD-RISC-10)

This scale was originally developed by Connor and Davidson (58) to measure resilience. Jeon et al. (59) validated the scale in a Korean sample, and the 10-item shortened form suggested by Campbell-Sills and Stein (60) showed the best fit. Therefore, the 10-item shortened form was used in this study. All items are scored on a 5-point Likert scale. The internal consistency (Cronbach’s α) for all items in this study was 0.91.

### 2.5 Analysis

The R programming language was used for data analysis. The participants’ sex, age, and level of burnout symptoms at the time of recruitment were examined, and the pretest homogeneity of the participant groups was tested. Differences in the sex distribution were analyzed using a χ^2^ test, and the pretest homogeneity of the main variables was tested using analysis of variance (ANOVA). Next, a mixed repeated-measures ANOVA was employed to test whether changes in the dependent variables with the treatment condition (group) and measurement time were significant. After validating whether the main effects of the treatment condition and measurement time were significant and whether the interaction effects between the treatment condition and measurement time were significant, simple effects analysis was used to identify the specific differences between groups. Bonferroni’s method was used for post-hoc analysis of the repeated-measures ANOVA.

## 3 Results

### 3.1 Descriptive statistics and pretest homogeneity testing

**Figure 2:**
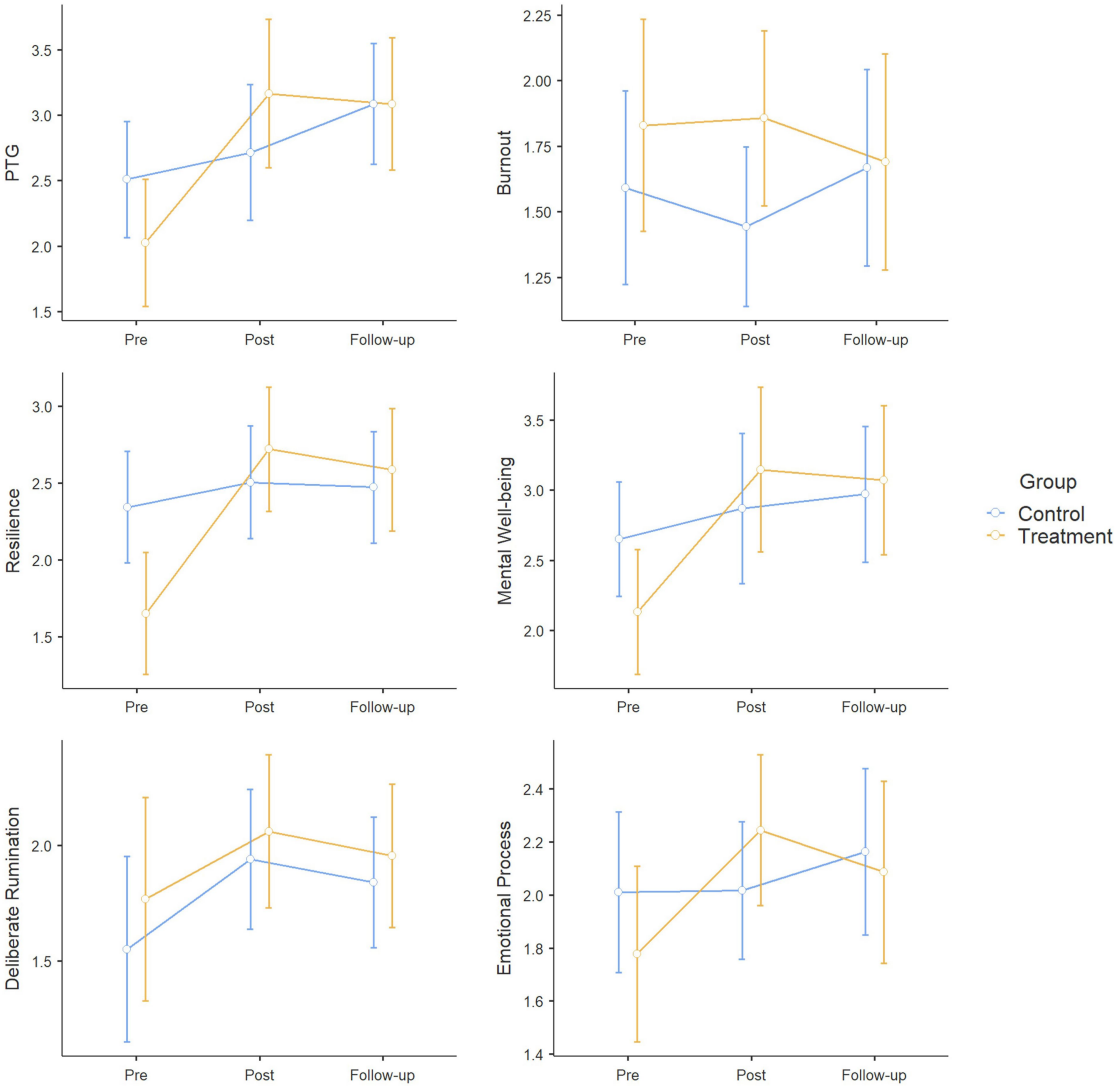
Change in the dependent variable by condition.

Descriptive statistics were calculated for all variables per treatment condition and measurement time point (Table 1). The variables were found to satisfy the assumption of normality based on the skewness, kurtosis, and QQ plots. Figure 2 illustrates the changes in the variables. The error bars in Figure 2 represent the 95% confidence interval of the mean measurement for each condition. No significant differences were found between the treatment and control groups in the sex ratio, *χ*^2^(1) = 0.503, *p* = 0.478, religious status, *χ*^2^(1) = 0.609, *p* = 0.435, or school year, *χ*^2^(3) = 2.414, *p* = 0.491. In the statistical comparison of pretest measurements between treatment conditions, resilience was the only variable that showed significant differences. In the pretest measurements, resilience was significantly lower in the treatment group (mean, 1.65) compared with the control group (mean, 2.34), *F*(1, 31) = 6.898, *p* = 0.013, *η*^2^ = 0.18.

**Table 1:** Descriptive statistics.

Dependent variables	Measurement	Control group (*n* = 18)			Treatment group (*n* = 15)		
		*M* (*SD*)	Skewness	Kurtosis	*M* (*SD*)	Skewness	Kurtosis
PTG	Pre.	2.51 (0.97)	0.38	0.09	2.02 (0.86)	0.75	−0.21
	Post.	2.72 (1.27)	−0.27	−1.58	3.17 (0.79)	0.25	−0.91
	Follow.	3.09 (1.05)	−0.50	−0.71	3.09 (0.84)	−0.60	−0.12
Burnout	Pre.	1.59 (0.72)	−0.20	−0.66	1.83 (0.82)	0.75	0.16
	Post.	1.44 (0.61)	0.58	−0.91	1.86 (0.65)	−0.31	0.23
	Follow.	1.67 (0.71)	0.11	−0.73	1.69 (0.85)	0.01	−1.21
Resilience	Pre.	2.34 (0.74)	0.12	−1.11	1.65 (0.77)	0.01	−0.33
	Post.	2.51 (0.81)	−0.56	−0.50	2.72 (0.71)	0.26	−0.74
	Follow.	2.47 (0.91)	−0.94	−0.19	2.59 (0.50)	−0.83	−0.16
Mental Well-being	Pre.	2.65 (0.84)	−0.28	−1.33	2.13 (0.85)	−0.18	−0.31
	Post.	3.24 (1.01)	0.06	−0.56	3.51 (1.12)	−0.84	0.58
	Follow.	3.17 (1.18)	−0.34	−0.46	3.58 (0.68)	−1.59	3.44
Deliberate Rumination	Pre.	1.55 (0.89)	−0.31	−0.81	1.77 (0.77)	0.48	−1.27
	Post.	1.94 (0.70)	−0.90	0.36	2.06 (0.53)	−0.29	−0.71
	Follow.	1.84 (0.67)	−0.62	−0.72	1.95 (0.48)	0.63	0.05
Emotional Process	Pre.	2.01 (0.58)	−0.19	−0.66	1.77 (0.68)	0.17	−0.30
	Post.	2.01 (0.45)	−0.67	−0.08	2.24 (0.62)	−0.95	0.30
	Follow.	2.16 (0.69)	−0.57	−0.58	2.09 (0.60)	0.02	−0.06

### 3.2 Testing differences between treatment conditions by measurement time

The mixed repeated-measures ANOVA was used to test whether there were significant differences in the dependent variables depending on the treatment condition and measurement time (Table 2). The Mauchly test of sphericity was performed to evaluate whether the repeatedly measured data across the three time points (pretest, posttest, follow-up) satisfied the assumption of sphericity, and all dependent variables were found to satisfy this assumption, *p* > 0.05.

**Table 2:** Results of repeated-measures ANOVA.

DV	Effect	Source	*F*	*df*_1_	*df*_2_	*p*	*η* ^2^	*η_p_* ^2^
PTG	Within	Time	14.744	2	62	< 0.001	0.12	0.32
		Time × Group	4.212	2	62	0.019	0.03	0.12
	Between	Group	0.002	1	31	0.967	0.00	0.00
Burnout	Within	Time	0.235	2	62	0.791	0.00	0.01
		Time × Group	2.423	2	62	0.097	0.01	0.07
	Between	Group	0.928	1	31	0.343	0.02	0.03
Resilience	Within	Time	11.934	2	62	< 0.001	0.11	0.28
		Time × Group	6.632	2	62	0.002	0.06	0.18
	Between	Group	0.321	1	31	0.575	0.01	0.01
Mental Well-being	Within	Time	8.483	2	62	< 0.001	0.08	0.21
		Time × Group	2.873	2	62	0.064	0.03	0.08
	Between	Group	0.028	1	31	0.869	0.00	0.00
Deliberate Rumination	Within	Time	3.861	2	62	0.026	0.04	0.11
		Time × Group	0.103	2	62	0.902	0.00	0.00
	Between	Group	0.605	1	31	0.443	0.01	0.02
Emotional Process	Within	Time	5.553	2	62	0.006	0.03	0.15
		Time × Group	4.126	2	62	0.021	0.02	0.12
	Between	Group	0.020	1	31	0.887	0.00	0.00

Within = Within-subjects effect, Between = Between-subjects effect, *η*^2^ = Partial eta squared.

First, regarding PTG, the main effect of measurement time, *F*(2, 62) = 14.744, *p* < 0.001, *η*^2^ = 0.12, and the interaction effect between treatment condition and measurement time, *F*(2, 62) = 4.212, *p* = 0.019, *η*^2^ = 0.03, were significant. In post-hoc analysis, compared with the pretest treatment group, the posttest treatment, *t*(31) = 4.433, *p* = 0.002, follow-up control, *t*(31) = 3.231, *p* = 0.044, and follow-up treatment groups, *t*(31) = 4.158, *p* = 0.004, showed significantly higher PTG.

Second, in terms of burnout, the main effect of measurement time, *F*(2, 62) = 0.235, *p* = 0.791, the main effect of treatment condition, *F*(1, 31) = 0.928, *p* = 0.343, and the interaction effect between treatment condition and measurement time, *F*(2, 62) = 2.423, *p* = 0.097, were all non-significant.

Third, regarding mental wellbeing, the main effect of measurement time was significant, *F*(2, 62) = 8.483, *p* < 0.001, *η*^2^ = 0.08. The interaction effect between treatment condition and measurement time, *F*(2, 62) = 2.873, *p* = 0.064, *η*^2^ = 0.03, was marginally significant. In post-hoc analysis, compared with the pretest treatment group, the posttest treatment, *t*(31) = 4.004, *p* = 0.005, and follow-up treatment groups, *t*(31) = 3.777, *p* = 0.010, showed significantly higher mental wellbeing. Among subfactors, for psychological wellbeing, only the main effect of treatment condition was significant, *F*(1, 31) = 5.489, *p* = 0.026, and post-hoc analysis revealed that the control group showed significantly higher psychological wellbeing than the treatment group, *t*(31) = 2.343, *p* = 0.026.

Fourth, regarding deliberate rumination, only the main effect of measurement time was significant, *F*(2, 62) = 3.861, *p* = 0.026, *η*^2^ = 0.04. In post-hoc analysis, compared with the pretest condition, deliberate rumination was significantly higher in the posttest condition, *t*(31) = 2.812, *p* = 0.025.

Fifth, with respect to emotional processing, the main effect of measurement time, *F*(2, 62) = 5.553, *p* = 0.006, *η*^2^ = 0.03, and the interaction effect between treatment condition and measurement time were statistically significant, *F*(2, 62) = 4.126, *p* = 0.021, *η*^2^ = 0.02. In post-hoc analysis, compared with the pretest treatment group, emotional processing was significantly higher in the posttest treatment group, *t*(31) = 4.210, *p* = 0.003.

Finally, regarding resilience, the main effect of measurement time, *F*(2, 62) = 11.934, *p* < 0.001, *η*^2^ = 0.11, and the interaction effect between treatment condition and measurement time were significant, *F*(2, 62) = 6.632, *p* = 0.002, *η*^2^ = 0.06. In post-hoc analysis, compared with the pretest treatment group, the posttest control, *t*(31) = 3.216, *p* = 0.046, posttest treatment, *t*(31) = 4.570, *p* = 0.001, and follow-up treatment groups showed significantly higher resilience, *t*(31) = 5.104, *p* < 0.001.

## 4 Discussion

We focused on burnout and mental health problems suffered by healthcare workers during the COVID-19 pandemic due to excessive workload. We developed and validated a program to prevent burnout and improve resilience in furue nurses by promoting PTG due to distress from the pandemic. The program increased many adaptive indices in the treatment group, and these effects persisted until the follow-up time point. Below, we summarize and discuss the main results of the study.

First, compared with the control group, the treatment group showed a significant increase in PTG from the pretest to the posttest condition, and the elevated PTG was maintained at follow-up. The first goal of this program was to encourage psychological growth in the participants through reflection on their own experiences of stress and hardships in relation to the COVID-19 pandemic. As intended, the program helped the participants in the treatment group to grow psychologically based on their own experiences of hardship. The increased PTG in the treatment group at posttest, when compared with the measurement in the control group, indicates a significant increase due to the treatment. Moreover, the PTG level in the posttest treatment group was maintained at follow-up, suggesting that the treatment effect is not transient and can be sustained in the long term.

In the early stages of the program, various elements including mindfulness were utilized to promote participants’ physical and emotional awareness and expression. Tedeschi and Blevins (61) explained that mindfulness plays an important role in the conversion of intrusive rumination to deliberate rumination in trauma survivors and promotes adaptive coping, such as positive reappraisal. In the psychological education sessions on burnout and stress, participants’ openness and sharing of their experiences would enable the experience of PTG by promoting the cognitive processing of traumatic events (62, 81).

The activities in the middle stages of the program deal with and validate the discomfort that participants feel when recalling past experiences. By encouraging participants not to avoid uncomfortable experiences, but to accept and find meaning in those events, these activities can increase psychological flexibility. In one study of disaster trauma survivors, lack of psychological flexibility and negative beliefs after trauma were associated with an increased risk of being classified in the high-risk group (63). Hence, these activities are expected to have played important roles in the participants’ positive changes. Furthermore, based on self-perception skills learned before the program, the participants in the treatment group not only had the opportunity to recognize their existing psychological resources in Session 6 and secure additional psychological resources but also to experience social support while interacting with the group members throughout the whole program. In Hobfoll’s (64) conservation of resources theory, people who have experienced stress lose their personal resources, and when these resources are depleted or insufficiently recovered, psychological problems occur. Moreover, Posttraumatic factors such as social support systems significantly affect the posttraumatic stress response (65). Participants in the treatment group experience validation of their own experiences and social support throughout the program. Appropriately processing one’s own negative experiences without avoiding the issue is important for recovery to an adaptive state (80).

In the activities in the later stages of the program, to encourage finding meaning in past experiences, the participants explored directions for life and important values and inspired hope that positive changes could be maintained in a process of mutual support among the group members. In a meta-analysis on the factors affecting PTG, ‘hope’ was the cognitive variable with the largest effect size alongside the centrality of events, which is the tendency to construct one’s life story based on traumatic events and related memories (66). Snyder (67) defined hope as a positive condition resulting from the interaction between pathway thinking, a process of thinking of methods to achieve one’s goals, and agency thinking, which is the motivation to move toward goals. These two elements of hope are essential for people suffering from a traumatic crisis, and people with the will to survive and the means to achieve their goals could be called hopeful people (68). Tedeschi and Calhoun (69) explained that hope provides a different perspective of one’s world that has been damaged by trauma. In addition, a future-oriented attitude can enable individuals to voluntarily make choices toward active goals and find meaning in life (70). Moreover, it can help with the strategic reconstruction of memories, which is necessary for recovery, by incorporating memories of past events into one’s personal narrative (71).

Emotional processing shows one mechanism of change in PTG. The level of emotional processing in the treatment group increased significantly from pretest to posttest compared with the control group, with these elevated emotional processing levels being maintained at follow-up. Avoidance of traumatic experiences (80) and negative appraisal of traumatic events (72) can interfere with the cognitive and emotional processing that is helpful for recovery. The improved emotional processing in the treatment group after the intervention indicates that the participants in this group had appropriately processed stressful experiences related to COVID-19 without showing avoidance. Meanwhile, deliberate rumination remained constant across all measurement time, irrespective of treatment condition. However, it is important to consider that time is required for intrusive rumination to be converted to deliberate rumination. The approximately 4-week long program might not have been long enough to improve the participants’ deliberate rumination.

Resilience also improved significantly in the treatment group compared with the control group, and the improvement was maintained at follow-up. In particular, resilience in the treatment group was significantly lower than in the control group at pretest, but increased to the level of the control group at posttest, and this was maintained at follow-up. Tedeschi et al. (81) highlighted that people who experienced PTG could show higher resilience later. To achieve PTG after a traumatic experience, self-reflection, a new understanding of oneself, and effective coping to deal with difficulties are required, and people who have experienced PTG commonly recognize new strengths within themselves. These changes can enhance resilience for future difficulties (73). According to reports during the program from participants who had performed hospital practice during the COVID-19 pandemic, there were many things to think about, such as wearing masks, and the increased workload acted as a stressor. Through this program, Improving the ability to cope appropriately with these difficulties could help when faced with similar difficulties in the future.

The level of burnout in the treatment group showed a decreasing trend in the follow-up condition compared with the pretest and posttest conditions, while the control group showed a relatively increasing trend at follow-up. Although this difference was not statistically significant, given the small sample size, this difference could still represent a meaningful result. We can predict that the treatment group participants, while participating in the program, were in the process of learning psychological factors to reduce burnout, and that these individuals would cope more effectively and recover even if they suffered stress after the program had been completed.

This study had several limitations and implications. The first limitation is that the treatment duration was not long. The program was originally designed to last 8 weeks, with one session per week, but due to the practical schedule of the participants, the program was completed in 4 weeks, with two sessions per week. As a result, the duration might have been insufficient to promote deliberate rumination and emotional control, which are known to be core factors for PTG. Another limitation is that we were unable to randomly allocate participants to the treatment and control groups due to the practical schedules of some participants. It is also possible that we were unable to derive statistically significant results for some tests due to the small sample size. For example, there was a small difference in burnout between the treatment and control groups in descriptive statistics, and in the follow-up condition, burnout in the treatment group showed a decreasing pattern, but this was not a statistically significant difference. Furthermore, the majority of participants in this study were female, and the small number of male participants makes it impossible to draw conclusions regarding gender differences. Although the nurses remain predominantly female, the number of male nurses has been steadily increasing worldwide (74). Therefore, to generalize the findings of this study over the long term, a sufficient number of male participants will be required.

This program was developed to provide psychological support for healthcare professionals, including nurses. To enhance its applicability in real clinical settings, it is necessary to further develop the program into an online format. During the course of this study, it became evident that although nurses were motivated to participate, real-world constraints made actual involvement difficult. Although some nurses were motivated to participate, their demanding shift schedules made it difficult to attend in-person, group-based sessions. These accessibility challenges underscore the limitations of traditional face-to-face psychotherapy, especially during times of heightened concern about infectious disease transmission. While online interventions pose challenges-such as limited therapist control, potential confounding variables, and difficulty in delivering immediate support-they also offer opportunities for innovation in design and implementation (75). These approaches may serve as a suitable alternative for populations, such as nurses, who are unable to access mental health care due to temporal and spatial constraints (76). Moreover, not only nurses but also individuals in other professions may experience burnout in certain disaster-related situations. Although this program was developed specifically for nurses, it could be expanded into a generalized program that can be applied to a broader population and address common disaster-related issues.

Despite these limitations, the research significance of this study can be explained as follows. In the process of overcoming the COVID-19 pandemic, healthcare professionals have highlighted the possibility of recurrence of a similar infectious disease outbreak, while also emphasizing the need for effective countermeasures (77, 78). Psychological support to prevent burnout and improve resilience is required for healthcare workers, including nurses, to prepare for future infectious disease outbreaks. The mechanisms for healing and growth developed in this study demonstrated potential effectiveness in supporting mental health in healthcare workers. Furthermore, the experience of psychological growth acquired through this program could provide practical help in preventing the stress and burnout that might be encountered in future healthcare settings.

This study extended the targets of mental health support beyond alleviation of psychological distress, aiming for an integrative approach of psychological growth and improvement of resilience. Sim and Im (79) showed that nurses during the COVID-19 pandemic simultaneously experienced psychological distress, including posttraumatic stress symptoms and PTG, and emphasized the need to view these experiences from a perspective that can integrate distress and growth. This integrative perspective can provide guidelines to enable psychological support professionals to more meticulously handle the difficulties experienced by nursing staff during epidemics. In this study, to verify the PTG-promoting effects of the program, we measured not only the outcome variable of PTG, but also deliberate rumination and emotional processing, which are important intermediate variables for PTG. By analyzing these intermediate variables, we were able to identify areas for improvement in the program. This provides evidence to enable continual revisions and improvements to this program through subsequent research. We anticipate that a more effective program will be realized and implemented in real-world settings in the future. Building upon these implications, the present findings underscore the relevance of PTG-based interventions as preventive strategies for mitigating burnout among nurses. By facilitating self-reflection, emotional processing, and the mobilization of psychological resources, the program provides a structured framework that can be incorporated into nurse training curricula, continuing professional development, and organizational wellness initiatives. At the institutional level, the integration of such interventions may contribute to the retention of nursing staff, alleviate the clinical and economic costs associated with burnout, and ultimately improve the quality of patient care. Furthermore, the adaptation of this program into scalable online formats may enhance accessibility and feasibility, particularly under conditions of heightened workload or future infectious disease outbreaks. Taken together, these findings offer not only theoretical insights into the mechanisms of PTG, but also practical guidance for developing sustainable, evidence-based preventive tools that safeguard the mental health of nurses and strengthen healthcare systems.

## Data Availability

The raw data supporting the conclusions of this article will be made available by the authors, without undue reservation.
