# Supplementary material for: Can fostering posttraumatic growth prevent burnout and promote resilience in future nurses?
Source: Front Public Health. 2025 Sep 30;13:1665351. doi: 10.3389/fpubh.2025.1665351 (PMC12518270; doi:10.3389/fpubh.2025.1665351)
Supplement: Supplementary file 1 [file Table_1.docx]

**Spplementary file 1. Program components**

The first topic was ‘group orientation and formation of group relationships.’ This topic was included in Session 1, and the goals were for the group members to understand the contents and method of the group program and form rapport with each other and with the group leaders. The main content for this topic consists of activities to relieve tension and promote a sense of closeness between the group members.

The second topic was ‘physical and emotional recognition and expression.’ This topic was divided between Sessions 1 and 2. The objective was to help the group members recognize and express their own physical sensations and emotions and recognize these feelings even during stressful situations or burnout. First, the participants practice mindful meditation and stretching for physical relaxation. The group members listen to an introduction to mindfulness and then perform stretching while focusing on their physical sensations and emotions. These activities are repeatedly practiced in Sessions 1 and 2. Next, the participants perform an activity where they use their bodies to express emotional words. These activities are mostly preparatory exercises that involve recognizing and expressing one’s emotions in a comfortable environment to provide a feeling of safety before proceeding with more profound cognitive tasks in subsequent sessions.

The third topic is ‘finding meaning in stressful events.’ This topic is included in Sessions 3 to 5. The purpose of this topic is for participants to reflect on how they think and what meaning they perceive in stressful situations. After receiving psychological education on stress and burnout, the group members share their experiences of feeling stress and burnout. They also reflect more deeply on their own experiences and try to change their previous thought patterns during burnout to a different mode. They reflect on what meaning they could derive from past stressful experiences. These activities can be viewed as the main cognitive task of the program.

The fourth topic is ‘finding happiness and value,’ which is included in Sessions 6 and 7. This consists of activities to find happiness and meaning in one’s life and to live life in a more constructive manner. Through various activities, the group members look for happy activities that they can enjoy, explore values that they consider important, and use these to set approximate directions for their lives.

The fifth topic is ‘inspiring hope and debriefing.’ This topic is covered in session 8. The group members set ultimate goals and plans for their lives based on what they have learned and experienced in the program so far. They hope to be able to live more positively in the future, and they encourage and support each other. Based on their personal values identified in previous sessions, they set more specific directions for their lives. They establish several intermediate and final goals for their lives and discuss potential obstacles and coping methods with other group members. In the final activity for the program, the participants create an artistic work to express their hopes for the future, messages of support and encouragement for themselves and other group members, and the elements that bring them happiness. This allows the group members to visually perceive the outcomes of the program. For this study, once the activities were completed, the participants underwent posttest measurements, received instructions about compensation for their participation, and the program was concluded.
