# Supplementary material for: Can fostering posttraumatic growth prevent burnout and promote resilience in future nurses?
Source: Front Public Health. 2025 Sep 30;13:1665351. doi: 10.3389/fpubh.2025.1665351 (PMC12518270; doi:10.3389/fpubh.2025.1665351)
Supplement: Supplementary file 2 [file Table_2.docx]

**Spplementary file 2. Demographics of participants**

|  | Treatment group | | Control group | | Total | |
| --- | --- | --- | --- | --- | --- | --- |
|  | n | % | n | % | n | % |
| Total | 15 | 45.45 | 18 | 54.55 | 33 | 100.00 |
| Sex |  |  |  |  |  |  |
| Female | 12 | 36.36 | 16 | 48.48 | 28 | 84.85 |
| Male | 3 | 9.09 | 2 | 6.06 | 5 | 15.15 |
| Hospital Practice |  |  |  |  |  |  |
| Yes | 9 | 27.27 | 9 | 27.27 | 18 | 54.55 |
| No | 6 | 18.18 | 9 | 27.27 | 15 | 45.45 |
| Religious |  |  |  |  |  |  |
| Yes | 7 | 21.21 | 6 | 18.18 | 13 | 39.39 |
| No | 8 | 24.24 | 12 | 36.36 | 20 | 60.61 |
| School Year |  |  |  |  |  |  |
| 1st and 2nd | 6 | 18.18 | 9 | 27.27 | 15 | 45.45 |
| 3rd and 4th | 9 | 27.27 | 9 | 27.27 | 18 | 54.55 |
